# IGHG1 induces EMT in gastric cancer cells by regulating TGF-β/SMAD3 signaling pathway

**DOI:** 10.7150/jca.56056

**Published:** 2021-04-19

**Authors:** Yuxuan Li, Pan Wang, Dongmei Ye, Xue Bai, Xuemei Zeng, Qiang Zhao, Zhiwei Zhang

**Affiliations:** 1 Key Laboratory of Cancer Cellular and Molecular Pathology in Hunan Province, Cancer Research Institute of Hengyang Medical College, University of South China, Hengyang, 421001, Hunan Province, China.; 2 Department of Pathology, Qingyuan People's Hospital, Qingyuan, 511500, Guangdong Province, China.; 3 Department of Pathology, The Third Affiliated Hospital of Nanchang University, Nanchang, 330008, Jiangxi Province, China.; 4 Department of Pathology, The First Affiliated Hospital of University of South China, Hengyang, 421001, Hunan Province, China.

**Keywords:** gastric cancer, IGHG1, migration and invasion, EMT TGF-β/SMAD signaling pathway

## Abstract

**Objective:** Gastric cancer is one of the most common malignant tumors in the world. IGHG1 is a differentially expressed protein screened out in gastric cancer in the early stage of the subject group. This topic explores the expression of IGHG1 in gastric cancer and the effect of IGHG1 on the proliferation, migration, invasion and EMT of gastric cancer SGC7901 cells and its mechanism of action.

**Methods:** Twenty cases of gastric cancer were purified by laser Capture Microdissection. The isotopic tags for relative and absolute quantification was used to label the proteins, and then analyzed and identified them by quantitative proteomics. Immunohistochemical staining method was used to detect the expression of IGHG1 protein in gastric cancer tissues. Western blot was used to detect the expression of IGHG1 in gastric cancer cells. The MTT and Petri dish clone formation experiment analyzed the effect of low expression of IGHG1 on the proliferation of SGC7901 cells. Scratch test and Transwell migration and invasion test to observe the effect of low expression of IGHG1 on the migration and invasion of SGC7901 cells. Western blot was used to detect the effect of low expression of IGHG1 on the expression of EMT-related proteins.

**Results:** 243 proteins related to gastric mucosal lesions were preliminarily identified. We found that IGHG1 is highly expressed in gastric cancer tissues compared with normal control tissues. IGHG1 promotes the proliferation, migration and invasion of gastric cancer cells. Compared with the control group, the expression of EMT-related proteins Vimentin, N-cadherin, TGF-β, P-SMAD3 was decreased and the expression of E-cadherin was increased after IGHG1 low expression.

**Conclusions:** IGHG1 induces EMT in SGC7901 cells by regulating the TGF-β/SMAD3 signaling pathway.

## Introduction

Gastric cancer (GC) is one of the most common malignant tumors in the world. The 2018 global cancer statistics show that from 2015 to 2018, there were more than 1 million new cases of gastric cancer and 783,000 deaths, ranking the fifth in the global incidence of malignant tumors and the third in the mortality rate of malignant tumors 1. The incidence of gastric cancer has gender differences, which the incidence of men is twice that of women. However, due to the degree of economic development and the difference in society and lifestyle, there are also obvious differences between different regions and different countries. In China, gastric cancer is one of the most common malignant tumors, and its morbidity and mortality rank second among all malignant tumors. The incidence of males is higher than that of females, and the age of onset of gastric cancer is mainly concentrated in 45-75 years old 2, which seriously endangering people's health and lives.

The IGHG1 gene is located on chromosome 14 q32.33, and the protein encoded by it is one of the subtypes of immunoglobulin IgG, which is located in the C region of the Igγ-1 chain 3. There are five different types of human immunoglobulins: IgM, IgD, IgG, IgE, and IgA 4. IgG is an important component of the adaptive immune system, accounting for 75-80% of the total Ig pool 5. IgG is composed of heavy and light chains, including α chain, δ chain, ε chain, γ chain and μ chain, and light chains including κ chain and λ chain. IGHG1 plays an important role in the occurrence and development of tumors. Its expression in ovarian cancer 6, 7, pancreatic cancer 3, prostate cancer 8, 9, papillary thyroid carcinoma 5 and other malignant epithelial tumors is significantly increased. It is involved in the pathological process of tumor cell EMT, proliferation, apoptosis resistance, immune escape and metastasis.

## Materials and methods

### Tissue samples

For LCM, 20 paired samples of normal gastric mucosa, atypical hyperplasia, poorly differentiated gastric adenocarcinoma, and lymph node metastatic carcinoma were taken from surgical resection of tumor specimens in the First Affiliated Hospital of South China University. None of the above patients received preoperative radiotherapy and chemotherapy.

### Laser capture microdissection

The target tissue was captured from frozen sections of gastric mucosal lesions using a Laser Capture Microdissection Microscope. Adjust the laser aperture, speed and intensity according to the distribution of field of vision, magnification and target tissue. In order to reduce individual genetic differences, all cut and purified tissues were from different tissues of 20 patients with gastric cancer. The purified tissues were classified and mixed for identification by iTRAQ labeling and two-dimensional liquid chromatography coupled with mass spectrometry.

### iTRAD labeling and LC-MS/MS analysis

Purified protein sample processed according to the manufacturer's protocol for iTRAQ reagent (Applied Biosystems). Dissolve the sample to be tested in 1 ml SCX buffer (25% v/v acetonitrile, 10 mM KH_2_PO_4_, pH 2.6), insert the sample solution to be tested into Polysulfoethyl column (2.1 mm×100 mm, 5 μm, 200 Å, The Nest Group, Inc.MA), Separation by 20 AD HPLC systematic chromatography, vacuum centrifugal concentration, dissolving with 50 μL RPLC A (5% CAN, 0.1% formic acid). The mixture was then separated on a C_18_ reverse-phase column (Zorbax 300SN-C_18_, 0.1×15 mm, 5 µm, 300 Å, 4.6×250 mm; microm, USA) at a flow rate of 300 µL/min for 90 min. he q-Star XL(Applied Biosystems, USA) system was used for MS/MS analysis.

ProteinPilot^TM^ software (Version 4.2, revision 1340) was used for database search. The mass spectrum data analyzed by Analyst QS1.1 (Applied Biosystems) is imported into ProteinPilot^TM^, choose Paragon Method for analysis.

### Case data

The tissue chip of gastric cancer was presented by Mrs. Xia Hong from the Cancer Institute of Nanhua University. Tissue microarray samples were collected from 90 cases of gastric cancer patients, including 20 highly differentiated adenocarcinoma tissues, 29 moderately differentiated adenocarcinoma tissues, 21 poorly differentiated adenocarcinoma tissues, 9 signet ring cell tissues, and 11 mucinous adenocarcinoma tissues. 70 normal gastric mucosa tissues from 90 gastric cancer (taken from normal gastric mucosa above 10 cm from the edge of the tumor). None of the above patients received radiotherapy or chemotherapy before surgery, and the diagnosis was confirmed by the pathologist.

### Cell line

Human immortalized gastric mucosal epithelial GES-1 cell line, human gastric cancer MGC803 cells, BGC823 cells and SGC7901 cell line were provided by the Tumor Research Institute of Nanhua University.

All the cell lines were cultured in a complete medium containing a mixture of 1640 medium (RPMI) and fetal bovine serum at a ratio of 9:1, and the culture flask was placed in a constant temperature and humidity cell incubator for routine culture.

### Antibodies and reagents used in the experiment

IGHG1 antibody was purchased from Abnova. TGF-β was purchased from Abcam, UK.E-cadherin, Vimentin, N-cadherin, SMAD3 and P-SMAD3 were purchased from CST USA. β-actin was purchased from Biosharp. Fluorescent secondary antibodies were purchased from LI-COR, USA. The IGHG1 interference carrier was purchased from PLL (Changsha Jiahe Biotechnology Co., Ltd.). RPMI 1640 cell culture medium and trypsin were purchased from Gibco, USA. The ready-to-use immunohistochemistry kit was purchased from Fuzhou Maixin Biotechnology Development Co., Ltd. DAB dyeing liquid was purchased from Kangwei Century Biotechnology Co., Ltd. Matrigel adhesive was purchased from Corning, USA.

### Immunohistochemistry (IHC)

The gastric cancer tissue chip was dewaxed to water and repaired with citric acid (P=6.0). Immunohistochemical staining was performed with reference to the instructions of the two-step assay kit (KIT-9710, Fuzhou Maixin Biotechnology Development Co., Ltd.). The IGHG1 antibody was diluted with PBS buffer at a dilution ratio of 5:50, DAB was developed, and hematoxylin was stained with nuclei.

### Plasmids and transfection

Three IGHG1 interference vectors were constructed. The target sequence information was CCAAGGACACCCTCATGAT and AGTGCAAGGTCTCCAACAA and GCTCCTTCTTCCTCTACAG, respectively, and the sequencing confirmed that the sequence is correct. The LipoMax transfection reagent transiently transfected the interference vector into SGC7901 cells.

### Immunoblot assay

Total cellular protein was extracted using a lysis buffer containing a protease inhibitor. The protein concentration was determined by the BCA method. Total protein was separated on an SDS-PAGE gel and transferred to a PVDF membrane. Skim milk is blocked and antibody-fed, using β-actin as an internal reference.

### Proliferation assay

Inoculate 5000 cells per well into 96-well plates, set 3-5 replicate wells, add 20 μL (5 mg/mL) of MTT solution to each well at 0 h, 24 h, 48 h, 72 h and 96 h after inoculation, avoiding light in the incubator Continue to culture for 4 hours. 150 μL of DMSO was added to each well, and the mixture was shaken for 10 min at a constant temperature oscillator (37 °C, 200 rpm), and a single wavelength of 570 nm was selected using a microplate reader to measure the absorbance.

### Colony formation assay

800 cells per well were inoculated into a 6-well plate, and a secondary well was set, and the culture solution was periodically replaced. When the cell mass was visible to the naked eye, the culture was terminated, the paraformaldehyde solution was fixed, and the crystal violet staining solution was stained, and the microscopic count was greater than 50 cell clones.

### Scratch assay

4-6×10^5^ cells per well were seeded in 6-well plates. When the degree of cell fusion reaches 90%, use 200 μL Tip head to scratch 1/4, 1/2 and 3/4 of the cell surface of the pore plate with a straight ruler. Take a picture and record the distance of the scratches at 0 h, 24 h and 48 h after the scratch.

### Transwell migration invasion assay

Matrigel and RPMI were formulated into working fluids in a ratio of 1:6. 50 μl of the working solution was added to the upper chamber of each chamber, evenly tiling, and incubated for 7 h (this step is not required for the migration assay). 200 μl (density 2×10^5^ cells/mL) cell suspension was added to the Matrigel-coated Transwell chamber. Fixed with paraformaldehyde 24 h later, stained with crystal violet staining solution. The number of invading cells was counted by three fields under the microscope.

### Statistical analysis

This study used paswstat and GraphPad Prism 5 software for statistical analysis. The data listed in this study are expressed as mean ± standard deviation, in which immunohistochemical staining results are statistically analyzed using Fisher's exact probability test; Western blot and cell clone formation assay results were analyzed by One-way ANOVA; MTT assay results were analyzed by Two-way ANOVA. All statistical analysis results showed that the difference was statistically significant at *P*<0.05.

## Results

### The differentially expressed proteins in different stages of gastric mucosa canceration were screened

In order to further understand the molecular mechanism of gastric cancer and obtain valuable diagnostic markers. The research group collected twenty matched gastric cancer tissues classified as normal gastric mucosa (NGM), atypical hyperplasia (AH), gastric poorly differentiated adenocarcinoma (GPDAC) and lymph nodes metastasis adenocarcinoma (LMGAC) for surgical excision of specimens. The above tissues were purified by Laser capture microdissection (LCM). Total proteins in different tissues were extracted and isotopically labeled. Through isobaric tags for relative and absolute quantification (iTRAQ) combined with two-dimensional liquid chromatography-tandem mass spectrometry (2D LC-MS/MS), the analytical chromatography of these different tissues was performed for quantitative analysis and detection. The expression of the identified protein was up-regulated when the quantitative ratio was greater than 1.5 and down-regulated when the quantitative ratio was less than 0.667. A total of 243 proteins related to gastric epithelial cancer were identified, 153 of which were up-regulated (Table 1) and 90 down-regulated (Table 2) in gastric cancer tissues. Among them, IGHG1 was up-regulated in gastric cancer.

### Expression of IGHG1 in gastric cancer and gastric cancer cells

**Figure 1:**
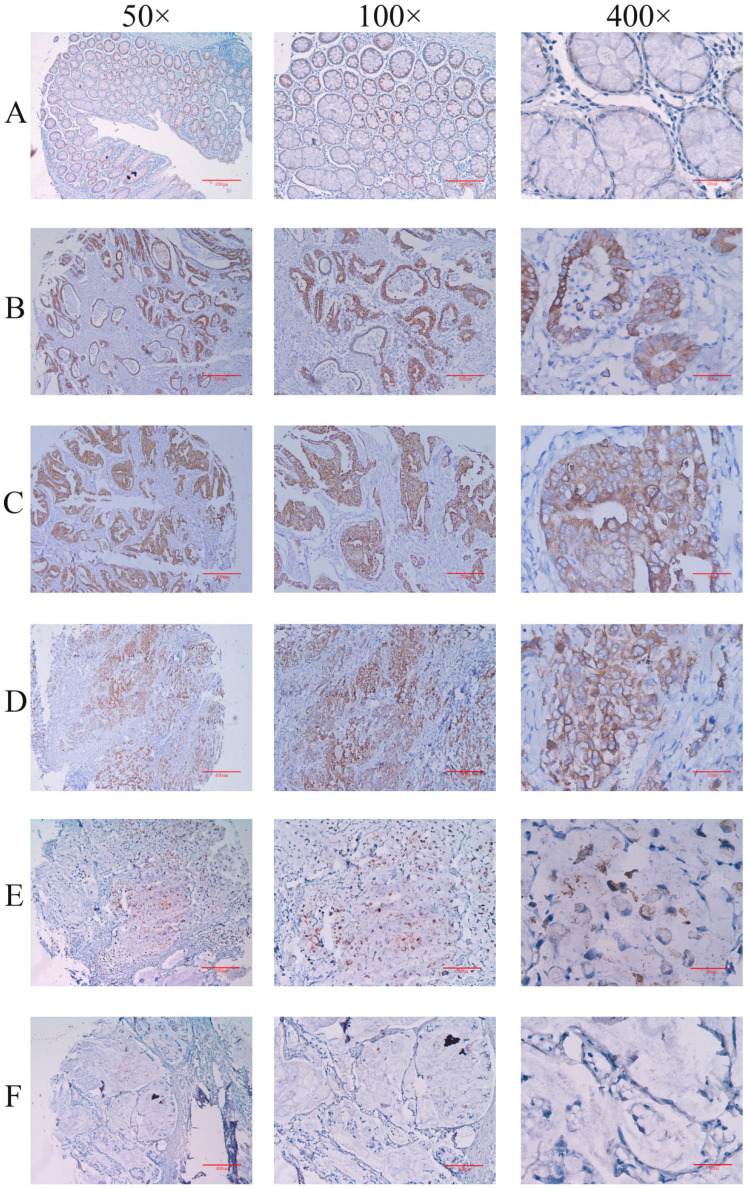
** Immunohistochemical method for detecting the expression level of IGHG1 in gastric cancer tissue microarray (A)** Normal gastric mucosal tissue. **(B)** Highly differentiated adenocarcinoma tissue. **(C)** Moderately differentiated adenocarcinoma tissue. **(D)** Poorly differentiated adenocarcinoma tissue. **(E)** Signet ring cell carcinoma tissue. **(F)** Mucinous adenocarcinoma tissue. **A-F**: Original magnification: 50×, scale bar: 400 μm; Original magnification: 100×, Scale bar: 200 μm; Original magnification: 400×, scale bar: 50 μm.

**Figure 2:**
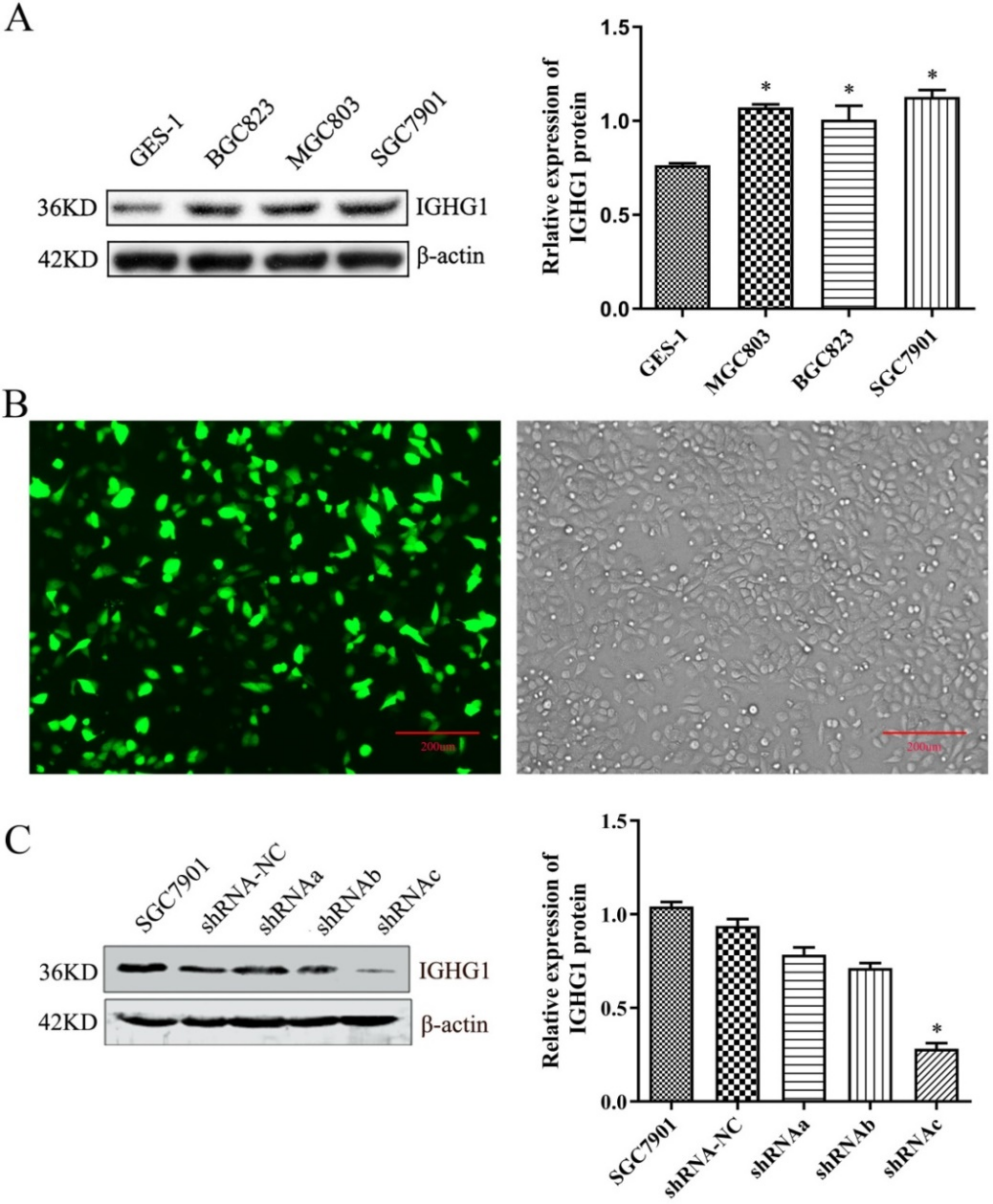
** Detection of IGHG1 expression level. (A)** Western blotting was used to detect the expression of IGHG1 protein in different gastric cancer cell lines (BGC823, MGC803, SGC7901) and immortalized gastric mucosal cells GES-1. IGHG1 was highly expressed in gastric cancer cell lines, and the highest expression was found in SGC7901 cells. **(B)** Fluorescent expression efficiency of gastric cancer cell SGC7901 after transient transfection of pPLK-shRNA-IGHG1. Original magnification, 100×. Scale bar: 200 μm. **(C)** Western blotting was used to detect the expression of IGHG1 protein after transfection of pPLK-shRNA-IGHG1 (shRNA-NC, shRNAa, shRNAb, shRNAc), and the expression level of IGHG1 protein was the lowest in shRNAc group. *Compared with the control group, *P*<0.05.

Firstly, the expression of IGHG1 protein in gastric cancer tissue chips (including 90 patients with gastric cancer) was detected by immunohistochemistry. The results showed that IGHG1 was mainly expressed in cell membrane and cytoplasm (Figure 1). The expression of IGHG1 in highly differentiated, moderately differentiated and poorly differentiated gastric adenocarcinoma tissues was higher than that in normal gastric mucosa tissues (*P*<0.05) (Table 3). Next, we examined the expression of IGHG1 protein in gastric cancer SGC7901, MGC803, BGC823 cells and immortalized gastric mucosal epithelial GES-1 cells by western blot. The results showed that the expression of IGHG1 in SGC7901, MGC803 and BGC823 cells was significantly higher than that in GES-1 cells (*P*<0.05, Figure 2A), and the expression level of SGC7901 cells was the highest. Therefore, we selected gastric cancer SGC7901 cells for subsequent interference experiments.

### Establishment of pPLK-shRNA-IGHG1 low expression cell line

The pPLK-shRNA-IGHG1 three-interference plasmid and the negative control plasmid were transiently transfected into SGC7901 cells, and the non-transfected group was set. After 24 hours, the transfection efficiency was observed under a fluorescence microscope. The results showed that the transfection efficiency reached 80%-90%, suggesting that the transfection efficiency was high and can be used in subsequent experiments (Figure 2B). Then, the total protein was extracted and the expression of IGHG1 protein in each group was detected by Western blot. The results showed that the pPLK-shRNAa-IGHG1 plasmid had an inhibition efficiency of 20%, the pPLK-shRNAb-IGHG1 plasmid had an inhibition efficiency of 30%, and the pPLK-shRNAc-IGHG1 plasmid had an inhibition efficiency of 70% (Figure 2C). Therefore, the shRNAc interference plasmid was selected for subsequent experiments.

### Effect of low expression of IGHG1 on proliferation of SGC7901 cells

**Figure 3:**
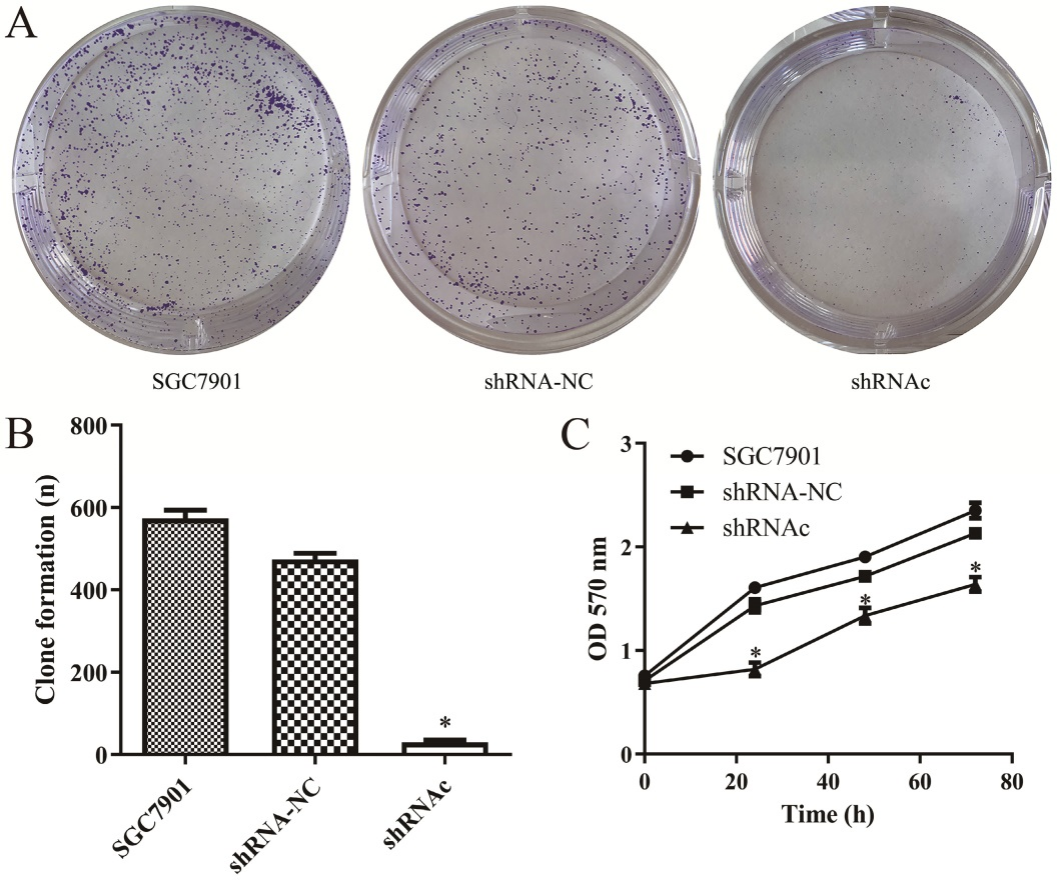
** Low expression of IGHG1 inhibits the growth and proliferation of gastric cancer SGC7901 cells. (A-C).** Colony formation and MTT assays in shRNA-NC, and shRNAc groups were transfected into SGC7901 GC cell line. *Compared with the control group, *P*<0.05.

In order to investigate the effects of IGHG1 low expression on SGC7901 cell proliferation, the MTT experiment was first performed. The results showed that the proliferation ability of SGC7901 cells was decreased after low expression of IGHG1 (*P*<0.05, Figure 3A-B). Then we conducted a plate colony formation experiment. The results showed that the cloning ability of SGC7901 cells was significantly decreased after low expression of IGHG1 (*P*<0.05, Figure 3C). In conclusion, the results indicated that the proliferative capacity of SGC7901 cells was decreased after low expression of IGHG1, suggesting that IGHG1 may be involved in the proliferation of SGC7901 cells.

### Effect of low expression of IGHG1 on migration and invasion of SGC7901 cells

**Figure 4:**
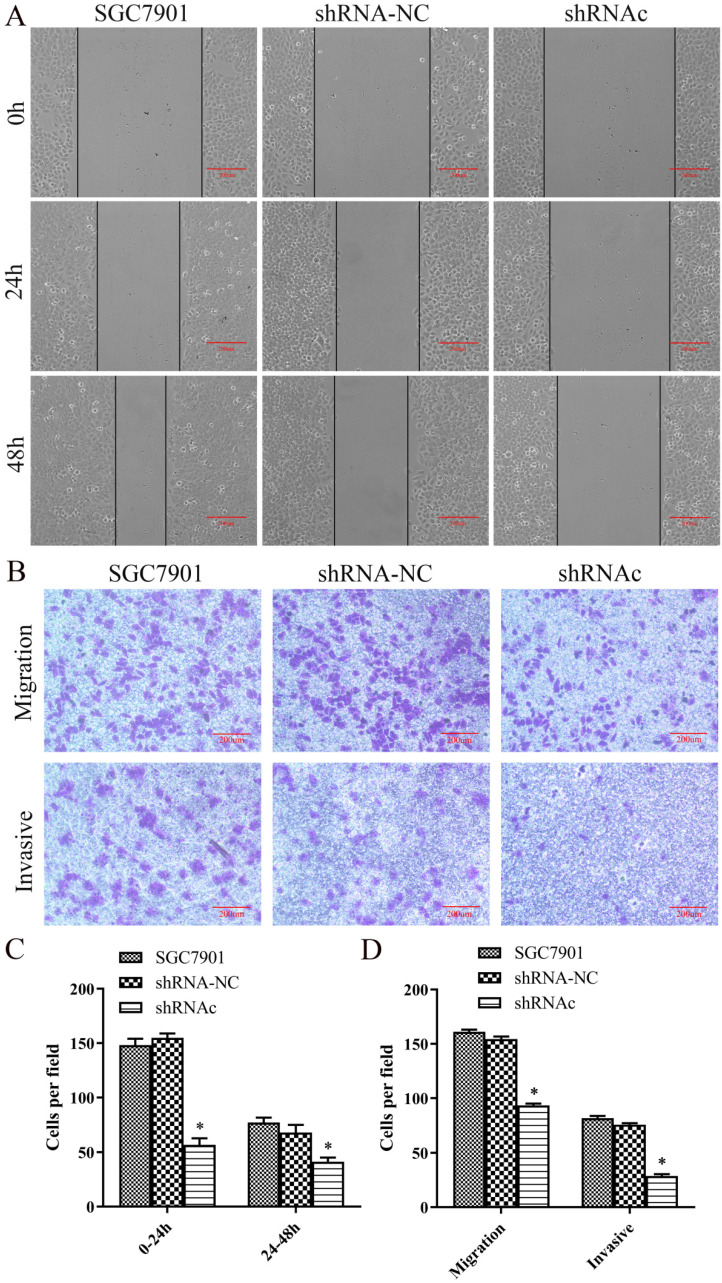
** Low expression of IGHG1 inhibits migration and invasion of gastric cancer cell line SGC7901.** SGC7901 cells were transfected with shRNA-NC and shRNAc, respectively. **(A)** Wound healing assay in the shRNA-NC, and shRNAc groups were transfected into SGC7901 GC cell line. **(C)** Migration index in the shRNA-NC, and shRNAc groups. Original magnification, 100×. scale bar: 200 μm. **(B, D)** Transwell migration and invasion assays in the shRNA-NC, and shRNAc groups. Original magnification, 100×. scale bar: 200 μm. Results are expressed as the mean ± SD with significance at *P<0.05. *Compared with the control group, *P*<0.05.

To understand the effect of low expression of IGHG1 on the migration of SGC7901 cells, we first performed a scratch test. The results showed that the migration distance of SGC7901 cells was significantly lower than that of IGHG1 (*P*<0.05, Figure 4A and 4C). Then Transwell migrated the invasion experiment showed that the number of migration and invasion of SGC7901 cells was significantly decreased after low expression of IGHG1 (*P*<0.05, Figure 4B and 4D). In conclusion, the results showed that the migration and invasion ability of SGC7901 cells was decreased after low expression of IGHG1, suggesting that IGHG1 may be involved in the migration and invasion of SGC7901 cells.

### Effect of low expression of IGHG1 on EMT-related molecules in SGC7901 cells

**Figure 5:**
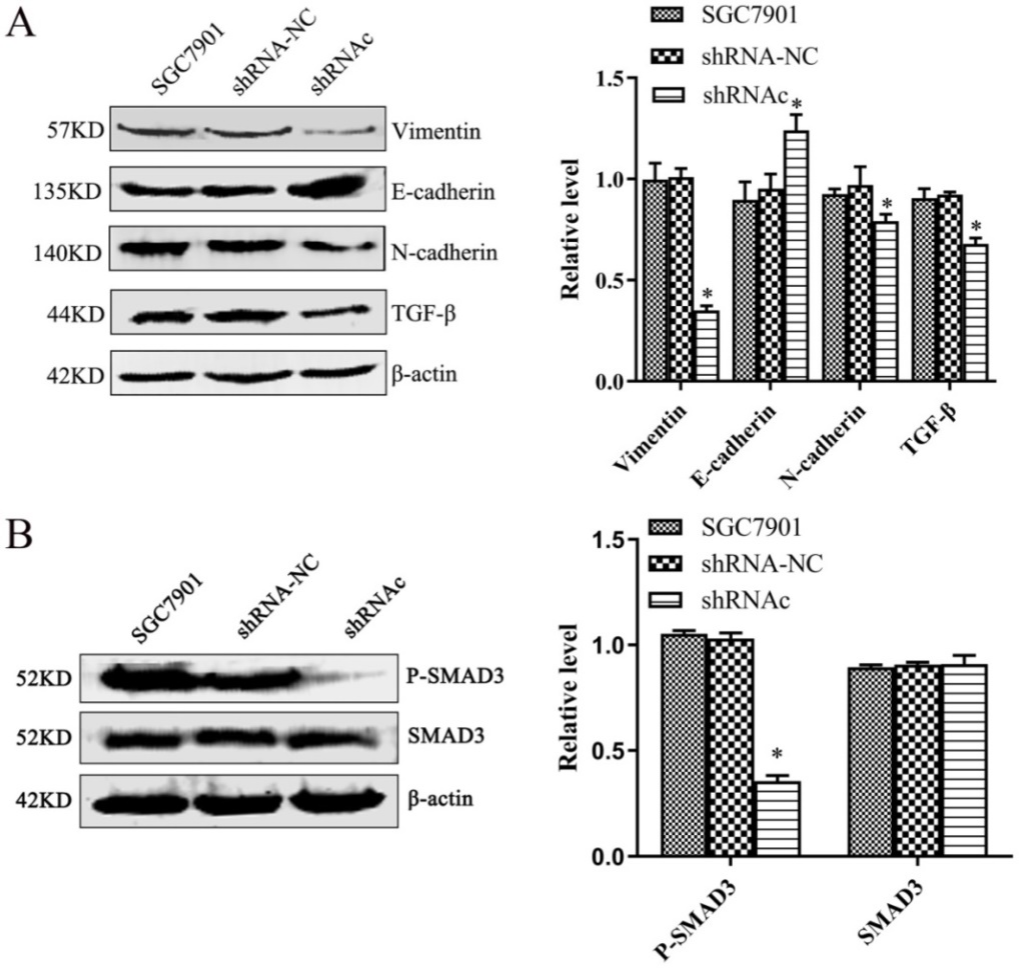
** Low expression of IGHG1 inhibits EMT (A)** Western blotting shows changes in the expression levels of EMT related proteins N-cadherin, E-cadherin, Vimentin and TGF-β in the shRNA-NC, and shRNAc groups.** (B)** Western blotting shows changes in the expression levels of SMAD3 and P-SMAD3 in the shRNA-NC, and shRNAc groups. Results are expressed as the mean ± SD with significance at *P< 0.05.

To explore the effect of low expression of IGHG1 on EMT of SGC7901 cells and its mechanism of action. We used western blot to observe the effect of EMT-related proteins after low expression of IGHG1. The results showed that the expression of N-cadherin, Vimentin and TGF-β was decreased and the expression of E-cadherin was increased in SGC7901 cells after low expression of IGHG1 (*P*<0.05, Figure 5A). The results showed that the ability of EMT in SGC7901 cells was weakened after low expression of IGHG1.

To further understand the effect of low expression of IGHG1 on the TGF-β signaling pathway. We used western blot to observe the changes of SMAD3 and P-SMAD3 after low expression of IGHG1. The results showed that the expression of P-SMAD3 in SGC7901 cells was decreased after low expression of IGHG1 (*P*<0.05, Figure 5B), but the expression of SMAD3 was not significantly changed (*P*>0.05). The results indicated that IGHG1 induced EMT in SGC7901 cells by regulating the TGF-β/SMAD3 pathway.

## Discussion

Gastric cancer is one of the malignant tumors with the highest morbidity and mortality in the world 1. The occurrence and development of gastric cancer is a complex pathological process involving multiple stages and multiple factors. Quantitative proteomic analyses can provide information for the search of potential biomarkers 10. At present, laser capture microdissection (LCM) is one of the best methods to purify tissue 11. Isobaric tags for relative and absolute quantification (iTRAQ) is widely used for differential expression and quantitative detection 12. The quadrupole time-of-flight tandem mass spectrometer (Q-TOF MS/MS) has high accuracy, high sensitivity and high resolution, which is conducive to the determination of extreme molecular weight, extreme PH and low abundance proteins 13. According to iTRAQ technology and 2D LCMS/MS, 243 differentially expressed proteins related to gastric cancer were preliminarily screened and identified, including IGHG1. It provides abundant material and experimental basis for search proteins in early diagnosis of gastric cancer.

The IGHG1 gene is located on chromosome 14, which is a protein-coding gene and is closely related to the occurrence and development of tumors. Immunoglobulin was traditionally thought to be produced by lymphocytes and plasma cells. Heavy and light chains of IgG have been detected in the cytoplasm of many human cancer cell lines 14. The study found that IGHG1 was highly expressed in tumors such as ovarian cancer, and participates in pathological processes such as EMT, proliferation, apoptosis resistance, immune escape and metastasis of tumor cells 6, 7. Studies have also found that IGHG1 was significantly more expressed in pancreatic cancer tissues than in non-cancer tissues, IGHG1 downregulates the cytotoxic effects of NK cells by inhibiting the antigen-dependent cytotoxic function, leading to proliferation and immune escape of pancreatic cancer cells 15. In current study, low expression of IGHG1 can inhibit the growth and induce apoptosis of prostate cancer cells 8. Its expression and mechanism of action in gastric cancer remains unclear. Based on previous studies, we found that IGHG1 was highly expressed in gastric cancer, participated in the proliferation, migration and invasion of SGC7901 cells and affects EMT of SGC7901 cells.

A large number of studies have found that the occurrence of EMT biological behavior plays an important role in the metastasis of malignant tumors 16-18. In gastric cancer, many signaling pathways were involved in the regulation of EMT, and signaling pathways such as PI3K/AKT 19, MEK/ERK 20, and WNT/β-Catenin 21, especially TGF-β/SMAD 22, 23 play important roles. Tumor cells transform from epithelial cells to stromal cells stimulated by TGF-β signaling was mediated by the SMAD pathway 24, 25. Members of the SMAD protein family are transcription factors that transmit TGF-β stimulation signals from cell membranes to the nucleus 26, 27. In our experiments, the expression of EMT signaling pathway protein TGF-β was decreased in SGC7901 cells when IGHG1 was poorly expressed, indicating that IGHG1 may promote the migration and invasion of gastric cancer cells through the TGF-β/SMAD signaling pathway. We found that the expression of P-Smad3 was significantly reduced after IGHG1 was poorly expressed, while the expression of SMAD3 was not significantly changed, which indicated that IGHG1 induced EMT in SGC7901 cells and promoted cell migration and invasion by regulating the activation of TGF-β/SMAD3 signaling pathway. Although IGHG1 interfered to reverse EMT occurrence in SGC7901 cells, there are still some shortcomings in this study. For example, this study only carried out follow-up analysis at the level of cell interference, and did not carry out *in vivo* experiments, which need to be verified by further experiments.

## Conclusions

This study found that IGHG1 induced cell migration and invasion by inducing EMT in SGC7901 cells by modulating the activation of the TGF-β/SMAD3 signaling pathway. The molecular mechanism of IGHG1 affecting EMT in gastric cancer SGC7901 cells was preliminarily confirmed, which provided experimental and theoretical basis for elucidating the molecular mechanism of gastric cancer.

## Figures and Tables

**Table 1:** Quantitative proteomics to identify up-regulated proteins in different stages of gastric mucosal epithelial carcinoma

No	Protein Name	AH *vs*. NGM	GPDAC *vs*. NGM	LMGAC *vs*. NGM	LMGAC *vs*. GPDAC
1	DDOST	↑2.559	↑1.803	↑2.831	↑8.017
**2**	**IGHG1 protein**	**↑1.690**	**↑2.421**	**↑4.245**	**↑2.291**
3	GPI Glucose-6-phosphate isomerase	↑2.673	↑2.805	↑1.677	↑9.201
4	PPIB peptidylprolyl isomerase B precursor	↑2.489	↑4.325	↑1.820	↑2.377
5	PRSS1 protein	↑4.966	↑4.446	↑7.253	↑3.076
6	RTN4 Isoform 1 of Reticulon-4	↑3.221	↑4.529	↑3.253	↑16.596
7	LGALS3 Galectin-3	↑7.112	↑6.918	↑2.466	↑2.805
8	Heat shock protein HSP 90	↑3.664	↑7.379	↑3.311	↑2.228
9	Myosin-9	↑4.525	↑7.586	↑3.342	↑2.270
…	…	…	…	…	…
145	TGM2	↑7.112	↑7.870	↑3.631	↑2.168
146	Secernin-1	↑1.959	↑7.870	↑1.660	↑4.742
147	HSP90B1 Endoplasmin precursor	↑4.742	↑8.395	↑1.995	↑4.207
148	type I cytoskeletal 17	↑2.228	↑8.710	↑2.333	↑3.733
149	Biglycan precursor	↑4.790	↑10.093	↑3.945	↑2.559
150	type I cytoskeletal 19	↑5.248	↑11.482	↑4.446	↑2.582
151	COMP Cartilage oligomeric matrix protein precursor	↑1.660	↑12.023	↑3.192	↑3.767
152	Cathepsin Z precursor	↑8.551	↑12.706	↑6.546	↑1.941
153	Collagen alpha-1(XII) chain precursor	↑4.267	↑14.997	↑4.366	↑13.804

No: Protein numbering; Protein Name: Protein name; AH *vs*. NGM, GPDAC *vs*. NGM, LMGAC *vs.* NGM, LMGAC *vs.* GPDAC Represents the ratio of protein expression between the two stages; NGM: normal gastric mucosa, AH: atypical hyperplasia; GPDAC: gastric poorly differentiated adenocarcinoma; LMGAC: lymph nodes metastasis adenocarcinoma.

**Table 2:** Quantitative proteomics to identify down-regulated proteins in different stages of gastric mucosal epithelial carcinoma

No	Protein Name	AH *vs*. NGM	GPDAC *vs*. NGM	LMGAC *vs*. NGM	LMGAC *vs*. GPDAC
1	Leucine-rich repeat-containing protein 59	↓0.655	↓0.142	↓0.273	↓0.520
2	Carboxyl ester lipase	↓0.249	↓0.191	↓0.461	↓0.413
3	chymotrypsinogen B2	↓0.550	↓0.236	↓0.525	↓0.449
4	Fibrinogen beta chain precursor	↓0.363	↓0.240	↓0.402	↓0.597
5	p180/ribosome receptor	↓0.555	↓0.242	↓0.614	↓0.394
6	SERPINA3	↓0.242	↓0.250	↓0.154	↓0.234
7	Coronin-1A	↓0.353	↓0.281	↓0.586	↓0.167
8	PRSS3 Isoform A of Trypsin-3 precursor	↓0.273	↓0.288	↓0.143	↑2.014
9	RPL14 protein	↓0.308	↓0.322	↓0.270	↓0.172
…	…	…	…	…	…
80	T-complex protein 1 subunit beta	↓0.319	↓0.380	↓0.013	↓0.483
81	Isoform 1 of Apoptosis-inducing factor 1	↓0.643	↓0.402	↓0.570	↓0.497
82	RPS27A 79 kDa protein	↓0.312	↓0.449	↓0.711	↓0.631
83	HNRNPA1 Isoform A1-B of Heterogeneous nuclear ribonucleoprotein A1	↓0.619	↓0.461	↓0.474	↓0.380
84	TSTA3 GDP-L-fucose synthetase	↓0.394	↓0.479	↓0.175	↓0.325
85	Calreticulin precursor	↓0.291	↓0.501	↓0.425	↓0.443
86	Heterogeneous nuclear ribonucleoprotein U	↓0.530	↓0.530	↓0.597	↓0.213
87	LOC100130562 hypothetical protein isoform 1	↓0.313	↓0.545	↓0.321	↓0.380
88	SLC9A3R1 Ezrin-radixin-moesin-binding phosphoprotein 50	↓0.406	↓0.592	↓0.149	↓0.560
89	IMMT Isoform 2 of Mitochondrial inner membrane protein	↓0.437	↓0.608	↓0.535	↓0.209
90	Mitochondrial inner membrane protein	↓0.437	↓0.608	↓0.535	↓0.209

No: Protein numbering; Protein Name: Protein name; AH *vs*. NGM, GPDAC *vs*. NGM, LMGAC *vs.* NGM, LMGAC *vs.* GPDAC Represents the ratio of protein expression between the two stages; NGM: normal gastric mucosa, AH: atypical hyperplasia; GPDAC: gastric poorly differentiated adenocarcinoma; LMGAC: lymph nodes metastasis adenocarcinoma.

**Table 3:** Expression analysis of IGHG1 in gastric cancer

Histological type	n	Negative	Positive	IGHG1 [(n %)]	*P* value
normal	70	35	35	50.00	
highly differentiated	20	4	16	80.00	0.033*
moderately differentiated	29	3	26	89.65	0.000*
poorly differentiated	21	2	19	90.48	0.002*
signet ring cell	9	2	7	77.77	0.223
mucinous	11	6	5	45.45	0.964

*Compared with normal gastric mucosa tissue.
